# Ensuring equity of access to primary health care in rural and remote Australia - what core services should be locally available?

**DOI:** 10.1186/s12939-015-0228-1

**Published:** 2015-10-29

**Authors:** Susan L. Thomas, John Wakerman, John S. Humphreys

**Affiliations:** Centre for Remote Health, Flinders University and Charles Darwin University, Alice Springs, Australia; Centre of Research Excellence in Rural and Remote Primary Health Care, Bendigo, Australia; Flinders Northern Territory, Darwin, Australia; School of Rural Health, Monash University, Bendigo, Australia; Flinders University, Alice Springs, NT Australia

**Keywords:** Primary health care, Equity, Access, Resource allocation, Health service planning, Health policy, Rural, Remote

## Abstract

**Introduction:**

Australians in rural and remote areas experience poorer health status compared with many metropolitan residents, due partly to inequitable access to primary health care (PHC) services. Building on recent research that identified PHC services which all Australians should be able to access regardless of where they live, this paper aims to define the population thresholds governing which PHC services would be best provided by a resident health worker, and to outline attendant implementation issues.

**Methods:**

A Delphi method comprising panellists with expertise in rural, remote and/or Indigenous PHC was used. Five population thresholds reflecting Australia’s diverse rural and remote geography were devised. Panellists participated in two electronic surveys. Using a Likert scale, they were asked at what population threshold each PHC service should be provided by a resident health worker. A follow-up focus group identified important underlying principles which guided the consensus process.

**Results:**

Response rates were high. The population thresholds for core PHC services provided by a resident worker were less in remote communities compared with rural communities. For example, the population threshold for ‘care of the sick and injured,’ was ≤100 for remote compared with 101–500 for rural communities. For ‘mental health’, ‘maternal/child health’, ‘sexual health’ and ‘public health’ services in remote communities the population threshold was 101–500, compared to 501–1000 for rural communities. Principles underpinning implementation included the fundamental importance of equity; consideration of social determinants of health; flexibility, effective expenditure of resources, tailoring services to ensure consumer acceptability, prioritising services according to need, and providing services as close to home as possible.

**Conclusion:**

This research can assist policy makers and service planners to determine the population thresholds at which PHC services should be delivered by a resident health worker, to allocate resources and provide services more equitably, and inform consumers about PHC services they can reasonably expect to access in their community.

This framework assists in developing a systematic approach to strategies seeking to address existing rural–urban health workforce maldistribution, including the training of generalists as opposed to specialists, and providing necessary infrastructure in communities most in need.

**Electronic supplementary material:**

The online version of this article (doi:10.1186/s12939-015-0228-1) contains supplementary material, which is available to authorized users.

## Introduction

Australians generally enjoy good health and experience one of the highest life expectancies in the world [1]. Unfortunately, however, many residents of Australia’s rural and remote communities experience poorer health outcomes compared with many of their metropolitan counterparts. Rates of potentially preventable diseases and avoidable hospitalisations increase significantly with geographical remoteness. Mortality rates for both males and females, possibly the best indicator of the health of the population, are significantly higher in very remote areas compared with major cities [2]. These outcomes reflect both the high proportion of socioeconomically disadvantaged and Indigenous residents with high disease burdens, and the inequitable access to primary health care (PHC) services for those living in rural and remote communities [1, 3]. Australia is not alone in striving for greater equity of access to PHC for those residing outside its urban areas. Both Canada and the United States, with their vast landscapes and scattered rural and remote communities, experiences similar health disparities that are linked to social determinants and poor access to PHC [4, 5].

Addressing this health disparity and the inequities in access to care requires a systematic national response. Considerable evidence exists to show that good PHC is associated with better health outcomes, lower costs and greater equity in health (reducing disparities across population subgroups) [6–9]. In 2009, the World Health Assembly urged countries to use national funding mechanisms to fast-track access to comprehensive PHC services that are equitable, efficient and sustainable [10]. Australia’s recent health reform process has sought to ensure that all Australians, including those in rural and remote areas, receive appropriate high quality and affordable primary and community health services [2]. This goal requires a more equitable distribution of resources and better access to comprehensive PHC services [11]. The National Rural Health Alliance reports an annual ‘rural health deficit’ of approximately $2.1 billion. This reflects an underspending on doctors, dentists and pharmacists in rural and remote communities, and an overspending of $829 million on hospitals in responding to the unmet PHC needs of rural and remote residents [12]. In short, Australia’s PHC system needs to boost its capacity to provide a range of basic services to Australians in rural and remote communities. Exactly how this will occur in not clear. However, the focus of this paper in identifying specifically what PHC services should be locally available in different-sized communities provides a much-needed platform for planning these necessary improvements.

Policy makers face significant problems in ensuring equitable access to sustainable PHC services in rural and remote areas, including the lack of locally-available services, insufficient workforce, inadequate infrastructure, high costs and long distances [13]. In seeking to overcome these problems and thereby improve equity of access, some jurisdictions have formulated health strategies which refer to ‘core’ PHC services that should be available at different levels within their health services network [14, 15]. This process, aimed at ensuring PHC services are as locally accessible to residents as possible, will be strengthened by having an evidence-based framework (that delimits how the provision of PHC services might vary according to population size and location) to guide them.

Recent research in Australia has outlined a set of core PHC services that all Australians can expect to access regardless of where they live [16, 17]. Each core service included a list of ‘illustrative services’ that provide examples from the literature of the kinds of services that would be provided under the broader headings, with the exception of oral/dental health, where no examples were found. See Table 1. PHC policy makers and service planners now have an evidence-based framework to guide resource allocation underpinning the provision of PHC services, rather than merely relying on existing historical rationing practices.

**Table 1:** Core primary health care services with illustrative lists for an Australian context [15]

Care of the sick and injured	Public health/Illness prevention
24 h care including evacuation	Immunisation
Treatment of injury and poisoning	Communicable disease control
Pathology	Targeted/health promotion programs
Radiology	Screening programs
Provision of essential drugs	Youth programs
Patient advocacy	Well men’s and women’s services
	Advocacy
Mental health/Social, emotional well being	Rehabilitation
Counselling	Alcohol and other drug rehabilitation
Drug and alcohol treatment	After trauma
	Post-CVA (stroke)
Maternal and child health	Oral/Dental health
Ante/post natal care	
Child development checks	
Immunisation	
Sexual and reproductive health	Allied health services
Sexually transmitted infections and blood borne viruses	Aged care and disability services
Family planning	Palliative care
	Counselling/social work/family violence
	Audiology
	Dietetics
	Occupational therapy
	Physiotherapy
	Podiatry
	Speech pathology
	Psychology
	Optometry

A key issue remains, however, how best to deliver core PHC services for Australia’s rural and remote communities which are scattered over such a vast geographical expanse. Specifically, considering what is fair and reasonable in a relatively wealthy country such as Australia, what PHC services should residents of different-sized communities, located in different geographical locations, be able to access from resident health workers as opposed to some alternative means of delivery such as visiting or tele-health services? While this is difficult and relatively uncharted research, answers to this question can assist policy makers and service planners to plan PHC service provision more equitably, thereby improving access to core PHC services for residents of rural and remote communities.

In the absence of comprehensive national data or evidence relating to the health and cost-effectiveness of different modalities of providing PHC services, answers to this key question may be subjective. Responses are likely to vary according to the perspective of different stakeholders. Nonetheless, it is important to ascertain the extent to which some common agreement can be obtained about which core PHC services residents of different sized communities should be able to expect to be available locally.

## Method

A Delphi method was used to determine consensus among rural and remote health experts in relation to differing population thresholds at which each of the core PHC services should be provided by resident health workers. Based on successive iterations, email surveys were used, thereby allowing the participation of a wide range of experts from across Australia without them having to meet face-to-face [18, 19]. Email surveys are quick and easy to administer, provide simple means to communicate with panellists and can result in high quality data collection [20]. Importantly, too, email surveys allow the ready participation of many different stakeholders who are widely dispersed across large geographical areas, in contrast to the high costs and sometime reluctance to travel associated with engaging them face-to-face. Researchers collated and returned the results allowing panellists to re-evaluate and adjust their previous responses in light of those of their peers. Iterations continued until the group reached some consensus or level of saturation. In order to ensure privacy and confidentiality, the panellists’ identities were known only to the researchers. Resulting anonymity prevented dominance by any individual and allowed all opinions to be considered [21]. In pursuing this research, the Delphi method was considered the most appropriate as the subject is complex, opinions are varied and there is a scarcity of published literature on the topic.

The 28 member Delphi group used here was a subset of a larger Delphi group of 39 experts that had been engaged in previous research on the core PHC services [17]. This group, comprising panellists who had completed three iterations as part of the previous Delphi study, included experts in rural, remote and/or Indigenous PHC who had been identified with the assistance of a national expert advisory group. Selection of individuals was based on their knowledge, experience and length of time working in the field of rural or remote health. Attention was paid to ensuring wide representation from areas of policy, the academic community, clinical practice and consumer representation. All states and territories were represented and members of key rural and remote health organisations were included. Potential panellists received a participant information statement with a letter of invitation. Informed consent was implied as panellists completed the first survey.

The survey instrument built on previous research, using a list of core PHC services including examples (referred to as ‘illustrative lists’). Five population categories were devised for communities smaller than 5000 residents to reflect Australia’s diverse rural and remote geography and settlement patterns. Population cut-offs, based on previous research measuring access to PHC services [22, 23], were deemed sufficiently sensitive to enable participants to differentiate population thresholds in relation to need for, and requirements of, different PHC services. Communities with populations more than 5000 were not included as it was assumed that in Australia these have access to resident health workers for all core PHC services. PHC ‘services’ refer to those dealing with prevention, detection, treatment and rehabilitation provided to patients, families and communities [15].

Participants were asked to consider PHC services for both rural and remote settings. There exists a vast literature distinguishing rural and remote. For the purpose of this study, remote communities were described as communities with small populations, located at a considerable distance from larger centres, usually in sparsely populated regions. These communities often have a high proportion of resident Indigenous Australians and a high degree of isolation (ASGC categories 4 and 5). Rural communities, on the other hand, referred to those relatively larger and/or less isolated communities located in more densely populated regions, which tend to be closer to larger centres where more comprehensive services may be available, such as hospitals and visiting or resident specialists (these are non-metropolitan rural communities not in ASGC 4 or 5) [24].

Using a Likert scale, and five population categories for rural and remote communities below the 5000 upper limit (‘≤100’ , ‘101–500’ , ‘501–1000’ , ‘1001–3000’ , ‘3001–5000’), Delphi panellists indicated the population threshold at which they believed each of the core PHC services should be provided by a *resident* health worker. Panellists answered separately for both rural and remote communities. Panellists were asked to select their answers based on what was ‘fair and reasonable in Australia’, without regard to current workforce, infrastructure or fiscal constraints. In the absence of firm rules for defining a consensus [25], this study adopted the following:

- Strong consensus- ≥80 % of panellists agreed
- Moderate consensus- 60–79 % of panellists agreed
- No consensus- < 60 % of panellists agreed

Following the iterative survey process, a face-to-face meeting with Delphi group panellists was used in a final consensus process to clarify issues of uncertainty arising in the final survey results. At this meeting discussion first clarified any issues associated with underlying assumptions and principles in relation to rural and remote service delivery that may have guided participants’ answers. Secondly, discussion focussed on reaching a consensus on population thresholds for resident service providers for each of the illustrative core PHC services in both rural and remote communities of different sizes. During the face-to-face meeting notes were taken by three research facilitators. Emerging themes were individually developed and compared for consistency.

Surveys were developed using Survey Monkey® with results analysed using Microsoft Excel 2010®. Ethics approval was obtained from the Central Australian Human Research Ethics Committee (CAHREC 12–57).

## Results

Twenty eight experts were invited to participate in the first round of the Delphi group. Two survey rounds were completed between October 2012 and March 2013. Response rates were 93 % (26/28) and 92 % (24/26) in the first and second rounds, respectively (see Table 2).

**Table 2:** Categories of Delphi panellists with response rates for 2 surveys and 1 face-to-face meeting

Category of panellists expertise	Accepted invitation to participate and sent round 1 survey n (%)	Responded to round 1 n (%)	Sent round 2 survey n (%)	Responded to round 2 n (%)	Invited to face-to-face meeting n (%)	Attended face-to-face meeting n (%)
Policy/management	11 (39)	9 (35)	9 (35)	8 (33)	8 (33)	3 (25)
Clinician	6 (21)	6 (23)	6 (23)	5 (21)	5 (21)	2 (17)
Academic	7 (25)	7 (27)	7 (27)	7 (29)	7 (29)	4 (33)
Consumer representative	4 (14)	4 (15)	4 (15)	4 (17)	4 (17)	3 (25)
Total	28	26	26	24	24	12
Response rate		26/28 (93)		24/26 (92)		12/24 (50)

Initially, there was considerable variation of expert opinion for many services. For example a strong consensus was not reached for any services in rural communities with a population ≤1000 residents. For remote communities with a population of 501–1000 there was consensus only for ‘care of the sick and injured’ (excluding ‘pathology’ and ‘radiology’), ‘maternal and child health’ (excluding ‘ante/post natal care’) and ‘public health/illness prevention’ (but only ‘immunisation’). These results are provided in an additional file (see Additional file 1). Because of the wide variation of responses, a face-to-face meeting was organised to discuss population thresholds in more detail. The 24 participants who completed the second iteration were invited to attend a face-to-face meeting in August 2013, of whom 13 accepted. One was unable to travel on the day, resulting in 12 participants (50 %).

Participants first discussed any assumptions that may have influenced their choice of population thresholds in the surveys. Following some group discussion, several sought to reconsider their previous responses. Group discussion also clarified the focus on PHC services *per se* rather than on professional groups, since PHC services may be provided by a wide range of professionals in different geographical contexts. Moreover, discussion made clear that core PHC services refer to basic rather than specialised/technical services.

It was re-iterated that researchers were seeking answers based on what was ‘fair and reasonable’ in Australia. The principles relevant to ensuring equitable provision of core PHC services in rural and remote contexts in Australia were discussed and agreed upon. For this study, these explicit principles were:

- the fundamental importance of equity (meaning that any avoidable disadvantages in access confronting those with greater needs and poorer health outcomes should be addressed as a matter of priority);
- taking account of the social determinants of health;
- flexibility ( the need to consider cultural, demographic and epidemiological factors particularity in small remote communities where the proportion of the population that is Indigenous is high);
- ensuring resources are well spent and so providing value-for-money in a constrained fiscal environment;
- tailoring services to ensure consumer acceptability, especially in Indigenous communities;
- prioritising services according to identified need – both normative and felt needs; and
- providing services as close to home as possible.

**Fig. 1:**
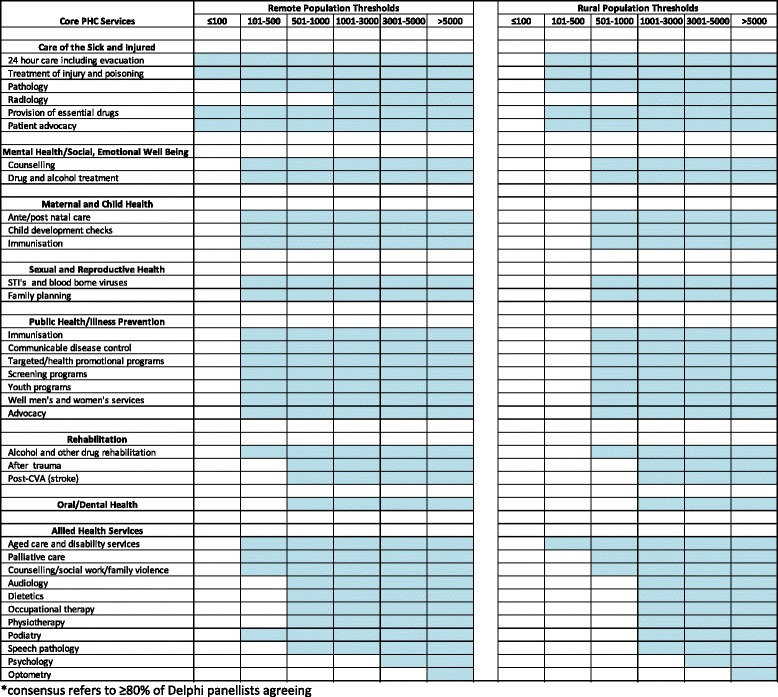
Final consensus amongst 12 Delphi panellists on rural and remote settlement size where primary health care services would be best provided by resident service providers*

In light of the discussion, the group then discussed their responses to the second round of the survey, and revisions were made. See Fig. 1.

Overall, the population thresholds were revised downward for both rural and remote communities, but population thresholds were still lower for services in remote compared with rural communities. Group members commented that because rural areas generally had better roads, smaller distances between settlements and better public transport, access to health services in larger settlements may be easier.

(i) *Rural communities*: For rural communities with populations over 100, experts agreed that most ‘care of the sick and injured’ and ‘aged care and disability’ services should be provided by resident health workers. There was a consensus that all services illustrative of ‘mental health and social and emotional well-being’ , ‘maternal and child health’ , ‘sexual and reproductive health’ and ‘public health/illness prevention’ as well as ‘counselling/social work/family violence’ , ‘palliative care’ , be provided by a resident health worker for communities more than 500 residents, while some core rehabilitation, oral health and allied health services exhibited a higher population threshold. The only service with a population threshold over 5000 was ‘optometry’.
(ii) *Remote communities*: For remote communities with a population of 100 or less there was a consensus that services illustrative of ‘care of the sick and injured’ (excluding ‘pathology’ and ‘radiology’) be provided by a resident health worker. There was a consensus that all services illustrative of ‘mental health and social and emotional well-being’, ‘maternal and child health’, ‘sexual and reproductive health’ and ‘public health/illness prevention’ as well as ‘counselling/social work/family violence’ , ‘aged care and disability services’ , ‘palliative care’ and ‘alcohol and other drug rehabilitation’ should be provided by a resident health worker in communities with populations above 100. While some core resident rehabilitation, oral health and allied health services exhibited a higher population threshold (501–1000) this was notably lower than in the case of rural communities. The only service with a population threshold over 5000 was ‘optometry’.

Qualifying comments were made in relation to some services. For example, for ‘care of the sick and injured’ there was agreement that communities with ≤100 residents needed to have some capacity for an emergency response (such as a locally-available first aid kit and a resident with first aid training) even if not provided by a resident health professional. For services such as ‘pathology,’ distinctions were made between taking a specimen and conducting the test. For ‘radiology’ the distinction was between taking an X-ray and the specialist skills of interpreting the results. For a number of ‘allied health’ services, it was commented that resident trained assistants could provide local care with support from a visiting allied health professional. Examples included an occupational therapist assistant working in a community of 500 residents, supported by an occupational therapist living in a settlement of 3000, or an oral health worker providing basic treatment and education in communities of 100 with a visiting dental service from a population centre of 5000 or more.

## Discussion

In the absence of comprehensive and rigorous empirical data, the task of defining population thresholds for the delivery of core PHC services inevitably relies on a degree of subjectivity based on underlying assumptions and principles that need to be explicit and agreed. Facilitating a face-to-face meeting as part of the Delphi process enabled consideration of, and agreement on, key assumptions and principles, as well as discussion of complex issues associated with reaching a strong consensus on the population thresholds.

Patterns of variation exhibited in relation to core PHC services were generally consistent across both rural and remote populations. Many of the services associated with ‘care of the sick and injured’ , ‘mental health and social and emotional well-being’ , ‘maternal and child health’ , ‘aged care and disability services’ , ‘palliative care’ , ‘sexual and reproductive health’ and ‘public health/illness prevention’ are best provided by resident service providers in communities with small populations. In contrast, many services included under ‘allied health’ , ‘oral/dental health’ and ‘rehabilitation’ required larger populations. These results accord with current health priorities in Australia associated with an ageing population, improving support for people living with disabilities, improving mental health services, addressing high rates of sexually transmissible infections in remote communities and the importance of preventive health strategies across communities. They also reflect recent government strategies described in the National Primary Health Care Strategic Framework, 2013, which aims to improve access to PHC, address service gaps and reduce inequity across Australia [26].

Importantly, population thresholds for almost all PHC services were notably smaller for remote compared with rural communities. This reflects their comparative geographical isolation, the nature and prevalence of health problems in many Indigenous communities, and the challenges in providing effective, sustainable PHC equitably in small communities where distances from larger centres are greater and transport less available.

It should be remembered that improving equity of access to core PHC services in rural and remote communities requires more than ensuring resident health workers are simply ‘on the ground’. Penchansky, for example, describes the five dimensions of access as availability, accessibility, accommodation, affordability and acceptability as important to achieving an optimal ‘degree of fit’ between health consumers and the health system [27]. Ensuring utilisation of PHC services is commensurate with community needs will require attention to all these dimensions [28]. For instance, acceptability is particularly important in communities with a high proportion of Indigenous Australians, where health services need to reflect community preferences, connection to culture and provide opportunity for self-determination [29].

Moreover, while there is an important role for consumers in the process of community planning, many of those in rural and remote settings (particularly those lacking comprehensive, sustainable PHC services) may not be aware of the range of PHC services that are available in metropolitan areas. Health planning for community needs must take into account community diversity and felt needs and be informed by knowledgeable consumer advocates and quality health data [30].

These findings ascertaining the population thresholds at which core PHC services would be best provided by resident health workers provide some guidance to policy makers and service planners tasked with the allocation of scarce resources for the provision of PHC services. They also help in developing a systematic approach to health workforce strategies which address gaps in service provision and the rural–urban maldistribution, including the training of generalists as opposed to specialists, ensuring professional support is available in small communities where maintenance of skills may be difficult, and for providing necessary infrastructure in those communities most in need. For policy makers, this study contributes to a systematic approach to strengthening PHC in communities most at need which is in the long-term more cost-efficient in preventing unnecessary hospital admissions [3–5]. Inequitable access to PHC and lack of evidence based frameworks useful in guiding resource allocation and service planning are not unique to Australia. Other countries such as Canada and the United States may benefit from adopting a similar framework as they struggle to address the misdistribution of health professionals and meet the basic health needs of residents in their rural and remote communities [4, 5].

Rapidly evolving technology may facilitate improved access for residents in some rural and remote areas through Point-of-Care Testing, tele-radiology, e-Health, tele-health and video-conferencing. However, given the concern firstly to maximise the access to, and availability of, local services provided by a resident health worker, such tools should be part of a broader strategy, and should not be relied upon as a substitute for solving the problems of PHC workforce undersupply or maldistribution.

This research acknowledges a number of potential limitations associated with use of a Delphi method. These include selection bias, small numbers of iterations and low response rates [19]. There are no set rules determining the number of panellists that will ensure validity of results, however 8–10 is thought to be adequate [31]. The panellists in this study were identified to ensure they represented rural and remote populations. Response rates were high and there was active engagement by all participants in the day long face-to-face meeting.

## Conclusion

Residents of rural and remote communities continue to experience poorer but avoidable health outcomes compared to many city residents. Defining the population thresholds at which core PHC services should be provided by resident health professionals is, firstly, an important step towards ensuring equity in access to health services and, more distally, in improving health outcomes. More equitable access to PHC services in rural and remote communities can undoubtedly contribute to reductions in rates of preventable diseases, avoidable hospitalisations and to lowering mortality rates.

## Additional file

Additional file 1:
**Second Delphi iteration; consensus on rural and remote populations where primary health care services should be provided by resident service providers*.** (DOCX 134 kb)
