# Supplementary material for: Ensuring equity of access to primary health care in rural and remote Australia - what core services should be locally available?
Source: Int J Equity Health. 2015 Oct 29;14:111. doi: 10.1186/s12939-015-0228-1 (PMC4625941; doi:10.1186/s12939-015-0228-1)

**Second Delphi iteration; consensus on rural and remote populations where PHC services should be provided by resident service providers***


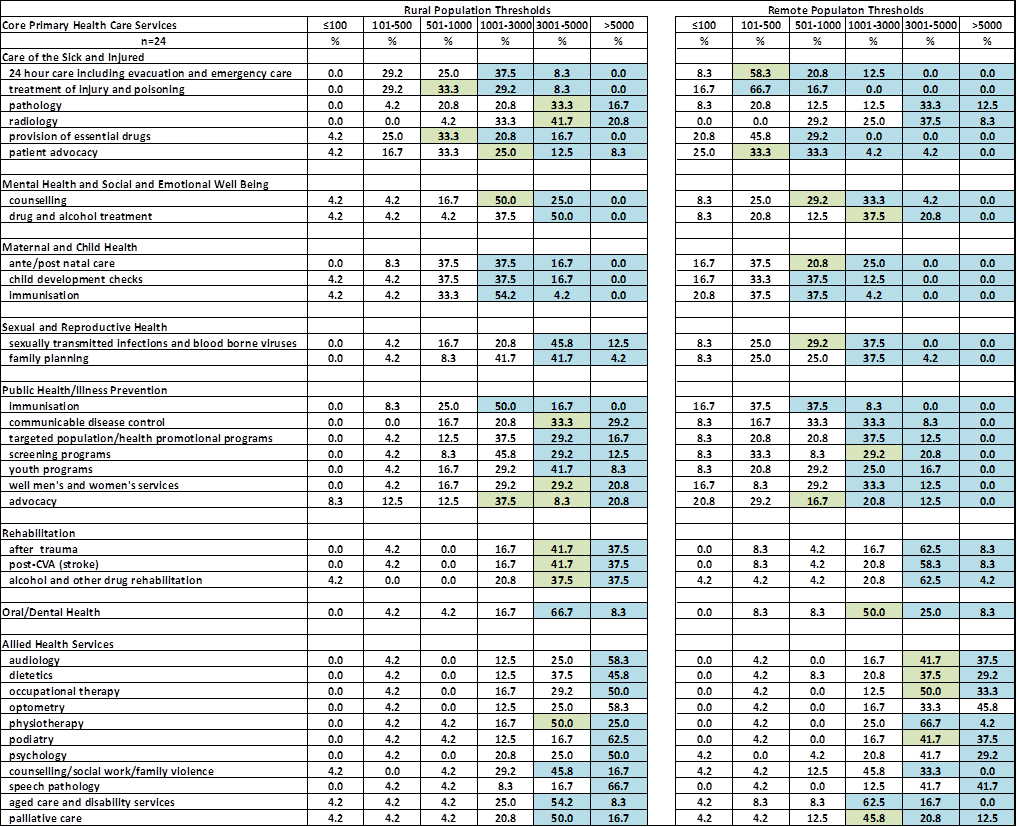


*survey results prior to face to face discussion


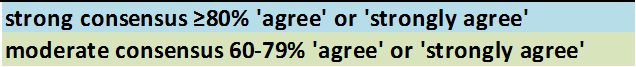

Supplement: Additional file 1: — Second Delphi iteration; consensus on rural and remote populations where primary health care services should be provided by resident service providers*. (DOCX 134 kb) [file 12939_2015_228_MOESM1_ESM.docx]
